# Transplantation of iPSC-TM stimulates division of trabecular meshwork cells in human eyes

**DOI:** 10.1038/s41598-020-59941-0

**Published:** 2020-02-19

**Authors:** Wei Zhu, Cheyanne R. Godwin, Lin Cheng, Todd E. Scheetz, Markus H. Kuehn

**Affiliations:** 1 Department of Pharmacology, Qingdao University, Qingdao, P.R. China; 2 Department of Ophthalmology and Visual Sciences, University of Iowa, Iowa City, IA 52242 USA; 3 Center for the Prevention and Treatment of Visual Loss, Iowa City Veterans Affairs Medical Center, Iowa City, IA 52246 USA

**Keywords:** Translational research, Molecular medicine

## Abstract

The trabecular meshwork’s (TM) physiological role is to maintain normal intraocular pressure by regulating aqueous humor outflow. With age, and particularly in eyes with primary open angle glaucoma, the number of cells residing within the TM is markedly decreased and the function of the tissue is compromised. Here we evaluate if transplantation of induced pluripotent stem cell derived TM like cells (iPSC-TM) restores TM cellularity and function in human eyes obtained from older human donors. Human iPSC were differentiated into iPSC-TM and compared to primary TM cells by RNAseq. iPSC-TM were then injected into the anterior segments of human eyes maintained in perfusion culture. Seven and 14 days eyes after injection eyes that received iPSC-TM contained significantly more cells in the TM. Fewer than 1% of all cells appeared to be iPSC-TM, but significantly more cells in these eyes were immunopositive for Ki 67 and incorporated BrdU. Our study demonstrates that transplantation iPSC-TM stimulates proliferation of endogenous TM cells in perfusion cultured human eyes from aged donors. These data, in concert with our previous findings in animal models, suggest that functional regeneration of the TM may be possible in human eyes with primary open angle glaucoma.

## Introduction

The aqueous humor in the eye is continuously replaced and is essential for many physiological functions, including provision of nutrients and removal of metabolic byproducts from the avascular tissues of the anterior segment. However, if outflow is perturbed the intraocular pressure (IOP) increases which can lead to cellular stress, dysfunction, and death of retinal ganglion cells resulting in vision loss^1–3^. This is frequently the case in glaucoma, a degenerative optic neuropathy affecting approximately 70 million people worldwide. Aqueous outflow from the eye to the Canal of Schlemm is controlled by the trabecular meshwork (TM), an avascular tissue located within the uvea and posterior to the corneal margin. The TM is populated by specialized cells and morphometric studies by numerous research groups have conclusively demonstrated that the cellularity of the TM declines with age and that it is particularly low in individuals with primary open angle glaucoma^4–8^.

Although a number of IOP-independent mechanisms contribute to loss of retinal ganglion cells in glaucoma, a reduction of IOP often slows or prevents vision loss in patients and approaches to reduce IOP either through medical or surgical means is currently the standard of care. However, surgical interventions are not without risk and voluntary or involuntary non-compliance with medical treatments of this chronic disease frequently hampers therapeutic success. Furthermore, most IOP lowering approaches either increase TM independent outflow or decrease the production of aqueous humor. This results in a reduction of aqueous humor flow through the avascular TM, potentially depriving the tissue of metabolites and further increasing cell stress. An alternative treatment approach has been the use of argon or selective laser trabeculoplasty, a procedure resulting in minor TM damage, but leading to TM cell repopulation^9^.

Based on the premise that replacing damaged or lost TM cells in glaucomatous eyes leads to prolonged functional restoration of the TM, we sought to develop a cell replacement approach that is translationally feasible. Ideally such cells should resemble TM cells in function, be easily obtained, and should be patient derived to enable autologous transplantation. Toward this goal we previously established methods to differentiate induced pluripotent stem cells (iPSC) into a cell type (designated iPSC-TM) that closely resembles native TM cells^10^. iPSC-TM express a number of TM cell markers, develop the ability to phagocytose material, and respond to exposure to glucocorticoids with elevated expression of myocilin and the formation of cross linked actin networks (CLANS)^10,11^. Transplantation of iPSC-TM into the eyes of mouse models of glaucoma results in an increased number of TM cells, enhanced aqueous humor outflow facility, decreased IOP and preservation of retinal ganglion cells^11,12^. In these animals we detected a significant increase in TM cellularity in iPSC-TM recipient eyes, but only a small fraction of these additional cells could be identified as iPSC-TM. Furthermore, transcriptional analyses demonstrated elevated transcript levels of numerous genes associated with cell division. These findings lead us to speculate that a main outcome of iPSC- TM transplantation is the induction of enhanced proliferation rates of endogenous TM cells.

The goal of this study was to demonstrate that iPSC-TM can also be generated from human iPSC. We further wanted to investigate whether these cells are capable of inducing a proliferation response in the TM of eyes obtained from donors of advanced age.

## Results

### Human iPSC-TM exhibit morphology resembling pTM

**Figure 1:**
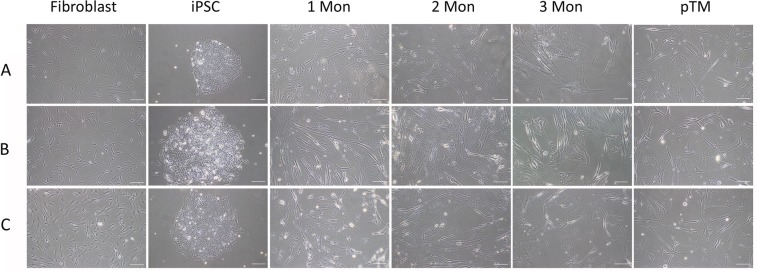
Morphological appearance of the human iPSC-TM cells during differentiation. Fibroblasts **(A**,**C**) or keratinocytes (**B**) were harvested from three distinct donors and pluripotency was induced (iPSC). Cells were then differentiated for one, two, or three months and compared to primary TM (pTM) cells from three unrelated donors (scale bar: 100 μm).

Dermal fibroblasts or keratinocytes were obtained from three donors and induced pluripotent stem cells (iPSC) were generated using methods described earlier^13,14^. These iPSC were then differentiated into a cell type resembling TM cells (designated iPSC-TM) through co-culture with human primary trabecular meshwork cells (pTM). As described earlier, iPSC maintained in this fashion begin expression of TM cell markers and acquire functional features of TM cells including phagocytosis and characteristic responses to glucocorticoids^10–12^. In this experiment the morphology of the differentiating iPSC was also monitored and compared to that of pTM cells. Within several days of co-culture, iPSC located at the edges of the colonies begin to elongate and increase in cell size (Fig. 1A). After 30 days of differentiation spindle shaped cells resembling pTM are frequently observed, but their size is typically smaller than that of pTM. In the days thereafter these pTM-like cells continue to increase in size until, after approximately 60 days of differentiation, they reach a size similar to that of pTM (Fig. 1). iPSC-TM can be maintained in culture for at least 120 days, but no additional morphological changes are noted past day 60. Although the rate of differentiation differed slightly between the iPSC-TM derived from the three donors, we did not observe any morphological differences between these independent cultures.

**Figure 2:**
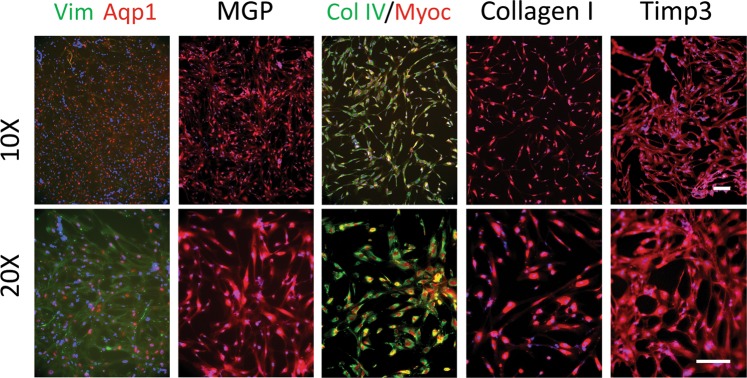
Immunohistochemical detection of TM cell markers in iPSC-TM shown at low (top) and high (bottom) magnification. Vimentin (Vim), aquaporin 1 (Aqp1), matrix GLA protein (MGP), collagen type IV (ColIV), myocilin (Myoc), collagen type I (Col I) and tissue inhibitor of matrix metalloproteinase 3 (Timp3) are readily be detected in iPSC TM from all three donors. (Scale bar: 200 µm).

The derived iPSC-TM also express a number of proteins characteristically associated with TM cells^15^, including vimentin, aquaporin1, matrix Gla protein, collagens I and IV, myocilin, and tissue inhibitor of metalloproteinases 3 (Fig. 2). These data suggest that the employed method of human iPSC-TM differentiation is robust and capable of generating a cell type strongly resembling pTM regardless of the iPSC donor’s genetic background.

### Molecular characterization of human iPSC-TM

To fully characterize the human iPSC-TM and their relationship to pTM, we determined their global transcriptional profile by RNAseq. iPSC-TM were harvested after 1, 2, or 3 months of differentiation and their transcriptional profile was compared to that of each donor’s original fibroblasts/keratinocytes, the derived iPSCs, and to those of pTM cultures established from three additional donor eyes.

**Figure 3:**
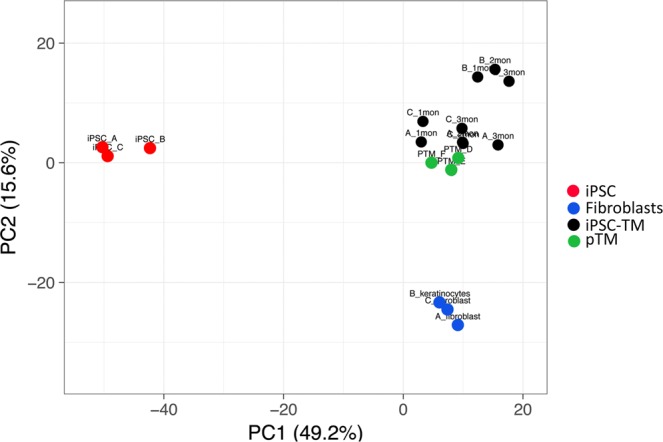
Transcriptional profiling using principal component clustering based upon the 2000 genes with the highest degree of variability. Samples derived from the original fibroblasts/keratinocytes (blue) and the iPSC derived from these (red) form distinct clusters than those representing iPSC-TM (black) or primary cultures of TM cells (green).

Based on the assumption that genes with little variability between samples are less likely to contribute to biological differences between cell types, we identified the 2000 genes with the highest expression variability based on standard deviation. Expression changes between the samples were investigated by Principal Component Analysis (PCA) (Fig. 3). These analyses reveal dramatic changes in the transcriptional profile as pluripotency is induced, and again during the differentiation of iPSC into iPSC-TM. iPSC-TM at all examined stages of differentiation clustered tightly with one another, indicating that the transcriptional signature of the cell is relatively stable throughout this period, despite apparent morphological changes. However, conserved transcriptional differences between pTM and iPSC-TM do exist. For example, iPSC-TM do not express the chemokines CXCL1, CXCL6, IL8, or CCL2 that are typically transcribed by pTM^16^.

iPSC-TM derived from fibroblasts (lines A and C) clustered tightly with pTM indicating that these cell types are very similar. Line B, derived from keratinocytes, clustered less tightly with pTM. Although the Euclidean distance between these two clusters is modest, it is indicative that multiple minor transcriptional differences exist between these cells and pTM. It is currently unclear if keratinocytes in general are less amenable to iPSC-TM creation or if the observed differences are specific to this particular line. Notwithstanding, iPSC line B was not used in the subsequent studies.

### Human iPSC-TM stimulate TM division *in vitro* and *ex vivo*

**Figure 4:**
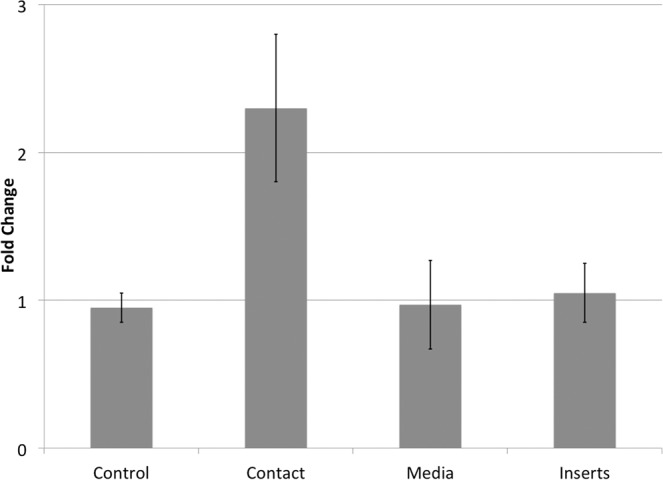
Normalized growth rates of TM5 cells following stimulation by iPSC-TM. TM5 cells grown in the presence of an equal number pTM (control), in iPSC-TM conditioned media (media), or when maintaining iPSC-TM in cell culture inserts (inserts) do not exhibit increased growth rates. In contrast, co-culture of TM5 and iPSC-TM allowing cell-cell contact (contact) results in a 2.3 fold increase in TM5 cell growth (p = 0.049, n = 3).

In our previous work, using mouse pTM and iPSC, we noticed that iPSC-TM exhibit a marked pro-proliferative effect on pTM. In order to determine that this effect can also be observed in human TM cells 20,000 fluorescently labeled cells from the TM5 cell line^17^ were co-cultured with either 20,000 purified human iPSC-TM cells or 20,000 pTM cells. After 4 days the number of TM5 cells was determined by flow cytometry. As expected, the presence of additional pTM did not influence the growth rate of TM5 when compared to controls (Fig. 4). Furthermore, culture of TM5 in cell culture medium previously conditioned by iPSC-TM or physically separating iPSC-TM by maintaining them in permeable tissue culture inserts, did not result in an increase in proliferation. However, co-culture with iPSC-TM allowing direct cell-cell contact increased the growth rate of TM5 by 2.3 fold (p = 0.0495, n = 3). These findings are in accordance with those previously observed using mouse cells and indicate that direct cell-cell contact is required for the pro-proliferative effect, suggesting that stimulation is not reliant upon secreted factors or exosomes.

We then evaluated the effect of iPSC-TM in an *ex vivo* human ocular perfusion organ culture system (POC). For this purpose the anterior segments of 12 pairs of human donor eyes were mounted in custom designed dishes and perfused at a rate of 2.5 µl/min. After 3–5 days, when the pressure within the cultures had stabilized, 200,000 GFP^+^ iPSC-TM cells were transcorneally injected into one eye, while the second eye from the same donor was maintained as a non-injected control. A sustained increase in outflow resistance as a result of the delivery of these cells was not observed. Eyes were harvested either 7 days (n = 5) or 14 days (n = 7) after transplantation, fixed in 4% paraformaldehyde and processed for histochemistry and immunohistochemistry.

**Figure 5:**
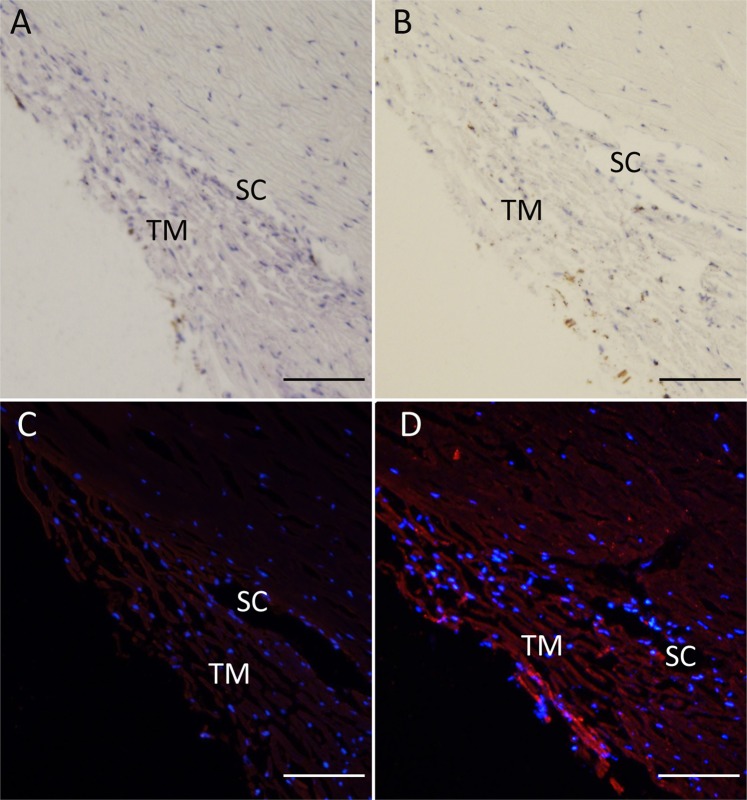
Effect of iPSC-TM transplantation on TM structure in the human ocular perfusion culture system. In sagittal sections no gross morphological changes are apparent in the TM of transplanted eyes (**B**,**D**) when compared to the contralateral untreated eye. (**A**,**C**) At this time iPSC-TM cells (pseudo colored red) are detectable, but rare, in transplanted eyes. (**D**) Nuclei are counterstained with DAPI to facilitate orientation. TM: Trabecular Meshwork, SC: Schlemm’s Canal, Scale bar = 100 μm.

Morphological assessment of the tissue indicated that delivery of iPSC-TM was well tolerated by all eyes (Fig. 5A,B). Two weeks after transplantation the TM of iPSC-TM recipients displayed organized beams and regular intertrabecular spaces, morphologically resembling the TM of vehicle control eyes. We then sought to examine iPSC-TM implantation into the TM but frequently encountered strong autofluorescence exhibited by the TM of older human eyes that prevented unambiguous direct visualization of GFP. Autofluorescence is much reduced in the far red channel and consequently we employed immunohistochemistry to detected GFP^+^ cells and visualized these with an AlexaFluor 680/790 (far red) secondary antibody. Using this approach GFP^+^ iPSC-TM implanted into the TM were evident in all transplanted eyes, although at low frequencies (Fig. 5C,D).

To determine the effects of iPSC-TM on TM cellularity after transplantation we employed morphometric methods similar to those used by other investigators^4–8,18^. 24 sagittal sections were obtained from at least two locations of the eye, stained with hematoxylin and eosin, and the number of nuclei located uveal and directly overlying Schlemm’s canal was counted. These numbers were normalized to represent the number of nuclei/100 µm. We also determined the number of implanted iPSC-TM using the same approach, but following immunohistochemical detection of the expressed GFP (Fig. 5).

As reported by others, the number of TM cells observed varied considerably between donors, ranging from 12.3 to 26.3 nuclei/100 µm (avg. 20.5 ± 7.8) in untreated eyes and from 22.9 to 45.7 nuclei/100 µm (average 32.6 ± 8.8) in iPSC-TM recipient eyes. In contrast, the number of cells observed in the two different regions of the same eye was highly correlated (R^2^ = 0.79, n = 24 eyes). As such, our data suggest that TM cell density within each eye does not vary considerably.

**Figure 6:**
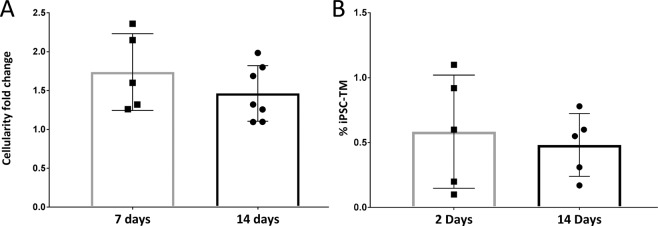
Effect of iPSC-TM on trabecular meshwork cellularity in human perfusion organ culture. (**A**) Transplantation of iPSC-TM resulted in a significant increase in the number of cells in the TM when compared to the untreated contralateral eye. This was apparent both one (1.8 fold) and two weeks (1.5 fold) after transplantation (p = 0.012 and 0.019, paired Student’s t-test). (**B**) RT-PCR based estimation of the fraction of cells containing Y-chromosomal DNA among all TM cells 2 and 14 days after transplantation. The fraction of Y-chromosomal DNA is indicative of the number of transplanted and surviving male iPSC-TM in the TM of female recipient eyes.

Studies by Tschumper and Johnson established that despite inter-donor differences in TM cell density, the cellularity between both eyes of the same donor is highly correlated^18^. Based upon these findings we analyzed the changes in TM cellularity between the untreated control eye and the contralateral iPSM-TM treated eye using a paired statistical test. Using this approach, we found that the total cellularity in iPSC-TM recipient eyes was notably higher than that in the contralateral control eyes in all cases (Fig. 6). This was evident as early as 7 days after transplant (average 1.76 ± 0.49 fold increase, p = 0.012) as well as after 14 days (average 1.46 ± 0.35 fold increase, p = 0.019).

However, as already noted above, the number of iPSC-TM in the TM was modest: 14 days after transplantation only 0.75% of all TM cells were GFP^+^, identifying them as iPSC-TM. One possible explanation for the paucity of GFP^+^ iPSC-TM could be that these cells decrease expression of this marker protein upon transplantation. Thus cells may be present, but undetectable by microscopy. To confirm our morphologic findings we carried out a molecular analysis using three pairs of eyes from female donors that had received transplants of iPSC-TM derived from a male cell line. The TM was dissected and DNA was extracted two days after transplantation from the first eye and 14 days after transplantation from the second eye. The amount of Y-chromosome DNA, indicative of iPSC-TM in the tissue, was then quantitated using a real-time PCR assay. Data obtained indicate that 2 and 14 days after transplantation only 0.6% and 0.5% respectively, of all cells in the TM contain Y-chromosomal DNA indicating that they are iPSC-TM derived from the male donor. These findings confirm our morphometric analysis and demonstrate that only a small fraction of cells residing in the TM are transplanted iPSC-TM.

**Figure 7:**
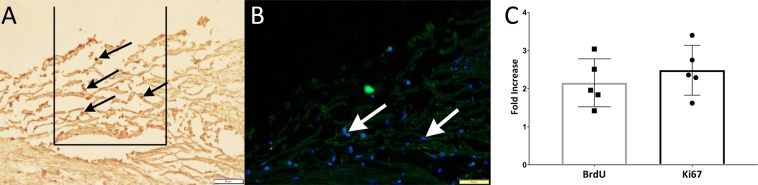
TM cell proliferation in human perfusion organ cultured eyes. Detection of (**A**) BrdU incorporation (black arrows) or (**B**) Ki-67 positive cells (white arrows) in sagittal sections of the TM seven days after iPSC-TM transplantation. (**C**) Quantitation revealed a 2.1 and 2.5 fold increase, respectively, in the number of dividing cells in iPSC-TM treated eyes when compared to untreated contralateral control eyes. The rectangle in (**A**) illustrates the TM area located uveal and overlying Schlemm’s canal used for morphometric analysis.

In the absence of considerable numbers of iPSC-TM contributing to the increase in total TM cellularity we hypothesized that, analogous to our observations in cell culture, transplantation of iPSC-TM stimulates proliferation of the recipients’ endogenous TM cells. To directly demonstrate cell proliferation we included BrdU in the perfusion media and maintained eyes for 7 days post-transplantation. BrdU is incorporated into newly synthesized DNA and can serve as a marker of cell replication. Using the morphometric approach described above, we determined the number of BrdU positive cells in the TM overlaying the TM (Fig. 7a). Our findings indicate higher numbers of BrdU positive nuclei in all iPSC-TM recipient eyes than in the contralateral controls (2.16 ± 0.61 fold, p = 0.0024). Lastly, we determined the density of KI-67 positive cells, an antigen that has been used extensively to identify cells in the G1, S, and G2 phase^19^ (Fig. 7b). In these same eyes, we detected a 2.48 ± 0.66 fold increase in the number of Ki-67 positive cells in iPSC-TM recipients when compared to the contralateral controls (p = 0.0047). Taken together, these data demonstrate that transplantation of iPSC-TM induces proliferation of human TM cells *in vitro* and in organ culture.

**Figure 8:**
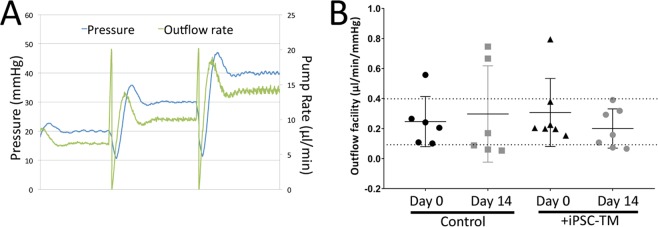
Effect of iPSC-TM on outflow facility in human perfused eyes. (**A**) Representative data illustrating the process of outflow facility measurements. Pressures of 20, 30, 40 mmHg (Blue line) are maintained by a computer controlled pump for one hour each. The required pump rates, corresponding to aqueous humor outflow, are recorded (green line). (**B**) Calculated outflow facilities in iPSC-TM treated and control eyes prior to transplantation (Day 0) and two weeks thereafter (Day 14).

Finally, we sought to functionally determine the effects of iPSC-TM transplantation in human POC. Aqueous humor outflow facility is a sensitive and dynamic parameter describing the functional state of the conventional outflow pathway^20^. It can be used clinically, but also in organ cultured eyes and we determined the outflow facility in iPSC-TM recipient and control eyes immediately prior to transplantation and 2 weeks thereafter (n = 7 pairs). Outflow rates were determined at pressures of 20 mmHg, 30 mmHg and 40 mmHg (Fig. 8). The outflow facility was then calculated based on the average outflow rate after the rates had stabilized, allowing for data collection for 30–45 minutes at each pressure.

During the 14-day period in which these normotensive eyes were maintained in organ culture we did not observe an increase in outflow facility in response to iPSC-TM transplantation. However, we did notice a stabilizing effect of iPSC-TM transplantation. In our hands, most normal human eyes exhibit outflow facilities between 0.2 and 0.4 µl/min/mmHg 4–5 days after establishing the organ culture system and the time iPSC-TM were transplanted. After two additional weeks in organ culture, the outflow facility often deviates from this range, presumably due to post-mortem decomposition of the tissue. In this experiment, 11 of 13 (84.6%) of all perfused eyes exhibited normal outflow facility prior to transplantation (5 of 6 in the control group and 6 of 7 eyes in iPSC-TM recipients, Fig. 8). Two weeks after transplantation – approximately three weeks in organ culture- only 1 of 6 eyes (16.7%) in the vehicle control group displayed normal outflow facility. In contrast 5 of 7 (71.4%) of eyes that received iPSC-TM exhibited normal outflow facility at that time (p = 0.048).

## Discussion

Lasting restoration of IOP control in patients with POAG holds the promise of significantly improving treatment outcomes. We have been exploring the use of iPSC derived TM-like cells for transplantation into the TM to achieve this goal. Our previous work, carried out in mice, demonstrated that iPSC from this species can be differentiated into iPSC-TM and that transplantation into the eyes of rodent models of glaucoma successfully lowers IOP and increases aqueous humor outflow facility. Herein we extend these studies and demonstrate that this cell type can also be generated from human iPSC using a similar protocol. Differentiation of human iPSC-TM proceeds at a slower rate than observed in mice, but the resulting cells are morphologically very similar to cultured primary human TM cells and express a number of TM cell markers.

We more fully characterized human iPSC-TM by comparing their transcriptional profile to pTM cells as well as the fibroblasts originally obtained from the donors. Principal component analysis demonstrates that iPSC-TM are clearly distinct from both the originating fibroblasts or keratinocytes and the resultant iPSC. Following differentiation, iPSC-TM and pTM form a relatively tight cluster indicating that their transcriptional activity is very similar. However, these cell types are not identical and a number of transcriptional disparities can be observed. Among these are presumably the, as of yet undefined, factors that give rise to the observed increase in proliferation of pTM cells. It is intriguing that a disproportionally large share of those genes expressed more abundantly in iPSC-TM than pTM is related to cell differentiation and proliferation. This may explain why a pro-proliferative effect can be observed when co-culturing pTM with iPSC-TM, but not when additional pTM are added. A second finding is that iPSC-TM derived from the same donor tended to cluster with one another, despite varying lengths of differentiation. This indicates not only that only small compositional changes occur after the first month of differentiation, but also that the genetic background of the donor affects the transcriptional profile of TM cells and that these individual differences are to some degree maintained by iPSC-TM. These findings are congruent with those published elsewhere demonstrating that individual differences among donors are a major cause of transcriptional variation among heterogeneous iPSC populations^21,22^. Variations in gene expression levels may complicate the interpretation of data, but on the other hand these findings also suggest that it may be possible to examine POAG associated TM phenotypes *in vitro* by creating iPSC based models of a patient’s TM cells.

The anterior segments of human donor eyes can be maintained in a Perfusion Organ Culture (POC) system, first reported by Johnson and Tschumper in 1987^23^. Use of this method allows for the investigation of cellular responses in their specific niche, but also enables the estimation of certain functional parameters, including outflow facility. In accordance with earlier studies^9,24,25^ we detected evidence of some cell proliferative activity in control eyes, but transplantation of iPSC-TM resulted in a significant increase in TM cellular density in the eyes of these older donors. All eye donors were of advanced age and perhaps due to the restricted age range a correlation between donor age and proliferative response could not be established, although there was a tendency for donors with fewer TM cells in the control eye to exhibit a larger proliferation response in the treated eye (data not shown). As previously observed in mouse models, the restoration of TM cellularity in the human eyes was not accompanied by broad implantation of iPSC-TM. We previously surmised that the increase is due to the proliferation of the recipients’ own TM cells and herein present data directly demonstrating the effect. Finally, we observed some incorporation of BrdU or KI-67 labeling in the untransplanted control eyes as well, supporting findings by others indicating that TM cells retain some proliferative ability throughout life^9,26^. Our data further indicate that the proliferative response of TM cells to iPSC-TM in human donor eyes occurs rapidly. TM cell proliferation was observed as early as 7 days after transplantation in perfusion organ cultures and no further increase was observed in the following week, indicating that the process was essentially complete after 7 days. This rapid proliferative response is congruent with our observations in the cell culture. Rapid induction of enhanced proliferation of mouse TM cells was also apparent in our previous studies and it is noteworthy that in these experiments functional improvement required several weeks. This suggests that aqueous humor outflow facility is not only related to the biomechanical activities of TM cells, such as contraction, but rather a slower process that occurs in the weeks following TM cell proliferation. One such process may be the maintenance of the TM extracellular matrix. TM stiffness increases with age and in POAG likely as a result of reduced ECM turn over due to lack or dysfunction of TM cells^27,28^. Thus one benefit of reestablishing TM cellularity may be the reversal of POAG associated ECM abnormalities, but this process is not likely to occur rapidly.

Despite the clear increase in TM cellularity, we did not observe an increase in outflow facility in iPSC-TM transplanted eyes. This is in contrast to our previous studies carried out in mouse models of glaucoma as well as those by others using human eyes with experimentally damaged TM^29^. However, there are at least two potentially important differences between these studies. First, the eyes used here were derived from human donors without elevated IOP, suggesting that the TM was still functional despite an age-related decline in cellularity. Thus an improvement in outflow facility might have been difficult to achieve in these relatively healthy eyes. Secondly, in our mouse model a significant increase in outflow facility was only achieved six weeks after transplantation, and the first signs of improvement are only evident after approximately four weeks. This time period is considerably longer than the maximum amount of time human eyes can be maintained in POC. In our hands, human eyes can be maintained for up to three weeks post-mortem and it is possible that this time period is insufficient for iPSC-TM to reverse degenerative changes in the TM. However, organ cultures of transplanted eyes were significantly less likely than control eyes to exhibit degenerative changes in the culture system. The aqueous humor outflow facility observed in the majority of eyes ranges from 0.1 to 0.4 μl/min/mmHg when POC is established; values similar to those reported in healthy human eyes^30^. After approximately three weeks in POC, a slight functional degeneration of the tissue is often observed. Accordingly, many of the untreated eyes exhibited either abnormally high or low levels of outflow facility, indicative of either outflow blockage or a disruption in the integrity of Schlemm’s canal endothelial cells, respectively. In contrast, most iPSC-TM transplanted eyes retained outflow facilities in the normal range until the end of the experimental period, suggesting a better functional status of the tissue.

It is encouraging that an increase in TM cellularity was observed in essentially all human eyes that received iPSC-TM transplants. The donor eyes used in this study were obtained from individuals with distinct genetic backgrounds and a variety of health conditions, including diabetes and age-related macular degeneration. This suggests that the observed proliferation response is likely to occur in most patients. However, eyes from donors with POAG were not included, which is a limitation of this study. Age related changes to the TM may, or may not, be similar to those occurring in glaucoma and it remains to be determined whether TM cells in POAG eyes will respond in a similar fashion as they may be uniquely damaged or their numbers may be too low in these patients. It is encouraging that TM cell stress and glaucomatous damage did not prevent a TM proliferation response and IOP decrease in a mouse model of glaucoma induced by expression of a variant of myocilin associated with juvenile glaucoma^11,31^ suggesting that TM cell therapy may be possible even in eyes with comparatively aggressive disease.

## Materials and Methods

### Human donor eyes

All donor eyes used for perfusion organ culture (POC) or the establishment of primary TM cultures (see below) were obtained from the Iowa Lions Eye Bank (Iowa City, IA) with written informed consent of the donor’s family. The experimental design was reviewed by the University of Iowa Institutional Review Board and determined to be exempt due to use of cadaver tissue. For POC 15 pairs of both male and female human eyes (age: 79.2 yrs ± 14.6) were collected within 12 hours post-mortem. None of the donors had a history of glaucoma, although other ocular disease including diabetic retinopathy and AMD were noted for some donors.

### Primary TM cell cultures

Briefly, the TM was dissected and treated with collagenase A (4 mg/ml) in DPBS for 2 hours. The tissue was then rinsed, centrifuged at 1,500× g and the cells contained in the pellet were maintained in Medium 199 (Gibco, MD, USA), containing 20% fetal bovine serum, heparin (90 ug/ml), endothelial growth supplement (Sigma, MO, USA), and 2 mM L-glutamine. Cells were maintained at 5% CO_2_ and 37 °C. Before experimentation pTM were validated following published recommendations^15^, including immunoreactivity to vimentin and absence of desmin expression and increased synthesis of myocilin upon dexamethasone treatment (see Supplemental Data).

### Human iPSCs differentiation

iPSC lines used were created at the University of Iowa following approval by the University of Iowa Institutional Review board. Written informed consent was obtained from all donors and all methods were carried out in accordance with relevant guidelines and regulations.

iPSC were derived from fibroblasts (lines A and C) or keratinocytes (line B) derived from 3 individuals without a history of glaucoma and reprogrammed using the CytoTune®-iPS Sendai Reprogramming Kit (Invitrogen, MA, USA), as described earlier^13,14^. iPSCs were seeded in Corning Synthemax coated plates and cultured in TeSR-E8 media until they reached 5–10% confluency. Primary TM cells (3^rd^–9^th^ passage) were seeded at 70–90% confluency in Transwell inserts (Corning, NY, USA) which were placed above the human iPSC. These co-cultures were maintained in biopsy media including MEM-α (Gibco, MA, USA), 10% fetal bovine serum (Gibco, MA, USA) and 0.2% primocin (InvivoGen, CA, USA) which was changed daily. The combination of primary TM cell culture and iPSC line was rotated every 10 days, ensuring that all iPSC lines were exposed to media conditioned by primary TM cells from all three donors. The morphology of all iPSCs, and iPSC-derived TM, was monitored under a bright-field microscope (Olympus, PA, USA).

### Transcriptional analysis

Total RNA was extracted from the original 4^th^ passage fibroblasts or keratinocytes, iPSCs, and the resulting iPSC-derived TM cells after 30, 60, and 90 days of co-culture using the QIAGEN RNeasy kit (Qiagen, CA, USA). In addition, RNA was obtained from 3^rd^ passage primary TM cells obtained from three eye donors. RNA quality was assessed using an Agilent BioAnalyzer 2100 (Agilent Technologies Inc, CA, USA). cDNA adhering to stringent QC measures was created by the TruSeq rapid kit (Illumina, CA, USA) for paired end reads. Stranded mRNA library was prepared for sequencing mRNA and generating the stranded mRNA information. 50 million reads were performed in primary TM D/E/F and 20 million reads were applied in the other samples. Sequencing was carried out on the Illumina HiSeq. 2500 sequencer (Illumina, CA, USA) as performed previously. Reads were mapped to the human genome (build hg19) using Tophat2 (ver. 2.0.11^32^. Isoform structure and abundance was calculated using Cufflinks (ver. 2.1.1)^33^. The fragments per kilobase per million mapped reads (FPKM) values for all genes were compiled from the Cufflinks output. In the subsequent analyses, genes with FPKM < 1 in all samples were removed. Samples were then clustered using singular value decomposition (SVD). Principal components analysis (PCA) was performed using ClustVis (https://biit.cs.ut.ee/clustvis/)^34^.

### Cell culture proliferation assay

Prior to experimentation human pTM cells were transfected with adenoviral vectors constitutively expressing the fluorescent protein dsRed (University of Iowa Vector Core Facility, Iowa City, IA). Then 20,000 pTM were co-cultured with either 20,000 human iPSC-TM cells or 20,000 unlabeled human pTM cells for four days. Alternatively, pTM were cultured in media previously conditioned by iPSC-TM for 24 hours, or iPSC-TM were maintained in permeable tissue culture inserts (0.4 µm pore size, Corning Transwell, Corning, NY). Cells were then harvested and incubated in a solution containing primary dsRed antibody (Abcam, MA, USA) and Alexa Fluor 546 conjugated donkey anti-rabbit IgG (Thermo Scientific, MA, USA) for 20 minutes at room temperature. After staining with Hoechst 33258 (Thermo Scientific, MA, USA), the number of dsRed^+^ TM5 cells was determined using a Becton Dickinson LSR II flow cytometer (BD, NJ, USA).

### Preparation of iPSC-TM for transplantation

After 60 days differentiation, 1 × 10^7^ iPSC-TM cells were trypsinized and suspended in 80 µl DPBS (Dulbecco’s Phosphate-Buffered Saline, Gibco, Gaithersburg, MD, USA) buffer with 0.5% Bovine Serum Albumin (BSA) (Sigma-Aldrich, MO, USA) and 2 mM EDTA (Sigma-Aldrich, MO, USA). 20 µl magnetic microbeads conjugated to Stage-Specific Embryonic Antigen 4 (SSEA-4) antibodies (Miltenyi Biotec, CA, USA) were added to bind cells retaining pluripotency. After incubating for 15 minutes at 4 °C, cells were washed twice with the DPBS buffer containing 0.5% BSA and 2 mM EDTA and loaded into a MACS LD column placed in a magnetic separator (Miltenyi Biotec, San Diego, CA, USA). After purification, cells were stained with R-Phycoerythrin (PE) conjugated anti SSEA-4 antibodies (BD Biosciences, NJ, USA) and incubated in the dark at room temperature for 20 minutes. Cells incubated with PE IgM isotype control antibody (BD, NJ, USA) were used as the control. After washing with DPBS and adding 2 µg/ml Hoechst 33258 stain (Life Technologies, NY, USA), samples were separated on a Becton Dickinson LSR II flow cytometer and assessed by DiVaTM software for the absence of cells expressing pluripotency markers (BD Biosciences, NJ, USA).

Cells were then transfected with HIV-CMV-GFP vector (University of Iowa Vector Core Facility, Iowa City, IA) at a multiplicity of infection of 5. After 6 hours cells were washed with MEF-alpha and cultured in fresh human pTM conditioned media for 10 days. Aliquots of these cells were then stored in liquid nitrogen. Three days prior to transplantation iPSC-TM were removed from storage and cultured in MEF-alpha.

### Human ocular perfusion system and measurements of outflow facility

Adult human donor eyes were briefly rinsed in a Povidone-Iodine solution. The anterior segment was dissected by placing a circumferential cut approximately 5 mm posterior to the limbus. After removing the remaining retina, the ciliary body and iris the anterior chamber carefully were rinsed in DEME media with 0.2% primocin (InVitrogen). The surface of the sclera was scored with a scalpel to allow outflow of the cell culture media through the episcleral veins. The anterior chamber was then placed into a custom made plexiglass dish and clamped with a flange and nylon screws. One port of this dish was connected to a 12 ml syringe containing DEME with 0.2% primocin which was loaded in a computer controlled syringe pump (World Precision Instruments, FL, USA). Eyes were perfused at a constant rate of 2.5 μl/min. A second port of the dish was connected to a pressure transducer whose output was recorded and analyzed using HemoLab software (Stauss Scientific, Iowa City, IA, USA). 3 mM 5-bromo-2′-deoxyuridine (BrdU) was added to the cell culture media of those eyes used for analysis of cell proliferation rates.

To determine the aqueous humor outflow facility, the pump rate required to maintain 20, 30, and 40 mmHg within the culture was determined. Briefly, the pressure within the organ culture is recorded every 5 seconds and analyzed by the Hemolab software. The software increases or decreases the pump rate in a stepwise fashion to maintain the target pressure and the required pump rate is recorded. Each pressure is maintained for one hour. Values obtained after the flow rate has stabilized are used to calculate the outflow facility (defined as µl/min/mmHg) by linear regression.

### Human iPSC-TM transplantation

2 × 10^5^ iPSC-TM cells in 30 µl perfusion media (DMEM with 0.2% primocin) were injected into the anterior chamber through the cornea using 33-gauge stainless steel needles. An equal volume of the perfusion media was injected into the fellow eye from the same donor. Eyes receiving iPSC-TM were randomly chosen.

### Morphometric analysis of TM cellularity

For all morphometric analyses anterior segments were fixed in 4% paraformaldehyde for 2 hours and embedded in OCT. A total of 24 sections (10 µm thick) from two distinct regions of the eye were obtained on a cryostat equipped with CryoJane tape transfer system (Leica Biosystem, IL, USA). These sections were either stained with Hematoxylin and Eosin (Sigma-Aldrich, MO, USA) or incubated with antibodies directed against KI-67 or BrdU as described below. Photomicrographs were taken at a magnification of 200x and used for quantitation. Values were obtained from all tissue located uveal to Schlemm’s canal and bordered by the anterior and posterior edges of Schlemm’s canal visible in the section. Counts were then normalized to represent the number of cells/100 µm.

For the detection of BrdU positive nuclei sections were rinsed and incubated in 10 mM sodium citrate at 95–99 °C for 15 minutes. Sections were allowed to cool and were then treated with 0.3% H_2_O_2_ in PBS for 10 min to deactivate endogenous peroxidase activity. They were then rinsed, blocked in 1% BSA and 0.3% Triton in PBS, and incubated with mouse anti-BrdU antibodies (1:500, Life Technologies, Thermo Fisher Scientific) in the blocking solution for 1 hour followed by incubation with goat anti-mouse horseradish peroxidase secondary antibody (1:500 in PBS) for 1 hour. After rinsing, DAB chromogen was applied and slides were incubated in the dark until the desired color intensity was reached.

KI-67 positive cells were detected following incubation of sections in 10 mM sodium citrate buffer at 85–95 °C for 20 minutes followed by incubation in blocking solution (1% BSA and 0.3% Triton in PBS) at room temperature for 1 hour. Sections were then incubated with rabbit anti-KI-67 antibodies (EMD Millipore, Billerica, MA) at 1:200 dilution for 3 hours. The secondary antibody was donkey anti-rabbit Alexa Fluor 488 (Life Technologies, Carlsbad, CA) was diluted 1:500 in PBS containing 4′,6-diamidino-2-phenylindole (DAPI, Life Technologies, NY, USA). Only nuclei positive for both KI-67 and DAPI were counted.

### Molecular assay for implanted iPSC-TM

For these experiments eyes from three female donors were used. iPSC-TM, derived from a male donor, were transplanted as described above. The TM was then harvested after 2 days from one eye and after 14 days from the fellow eye. DNA was extracted and the number of surviving iPSC-TM, based upon the amount of Y-chromosomal DNA detected, was determined using a quantitative PCR assay. For this samples were amplified using Y-chromosome specific primers (Forward: 5′-CAGATCCCGCTTCGGTACTC-3′, Reverse: 5′-TTTGTCCAGTGGCTGTAGCG-3′) in a BioRad CFX96 thermocyler (Hercules, CA) and compared to a standard curve. Values obtained were used to calculate the number of male iPSC-TM present based on the average weight of a human nucleus (7.2 × 10^−12^ g).

### Immunohistochemistry

Sections of 10 µm thickness were rinsed with DPBS for 3 times, blocked with 1% BSA and 0.3% triton for 1 hour, and incubated with GFP antibody (Abcam, MA, USA) overnight at 4 °C. After washing with DPBS for 3 times, Alexa Fluor 546 conjugated donkey anti-rabbit IgG (Thermo Scientific, MA, USA) and 0.1 µg/ml DAPI were used to stained nuclei for 1 hour at room temperature. Slides were sealed by using AQUA-Mount (Thermo Scientific, MA, USA) and dried at room temperature for 2 hours. Photomicrographs were taken on a fluorescence microscope (Olympus, PA, USA).

### Statistical analysis

TM cellularity, and number of BrdU or Ki-67 positive cells was evaluated between the treated and untreated fellow eyes from the same donor and p-values were calculated using a paired Student’s t-test. Differences in the frequency of eyes to maintaining normal outflow facility were evaluated using a Chi-square test.

## Supplementary information

Supplementary data.

## Data Availability

All manuscript related files and data are available from the corresponding author on request.
