# Supplementary material for: Transplantation of iPSC-TM stimulates division of trabecular meshwork cells in human eyes
Source: Sci Rep. 2020 Feb 19;10:2905. doi: 10.1038/s41598-020-59941-0 (PMC7031365; doi:10.1038/s41598-020-59941-0)
Supplement: Supplementary file 1 — Supplementary data. [file 41598_2020_59941_MOESM1_ESM.pdf]

Transplantation of iPSC-TM stimulates division of trabecular meshwork cells in human eyes

Wei Zhu[1], Cheyanne R, Godwin[2,3], Lin Cheng[2], Todd E. Scheetz[2], Markus H. Kuehn[2,3*]

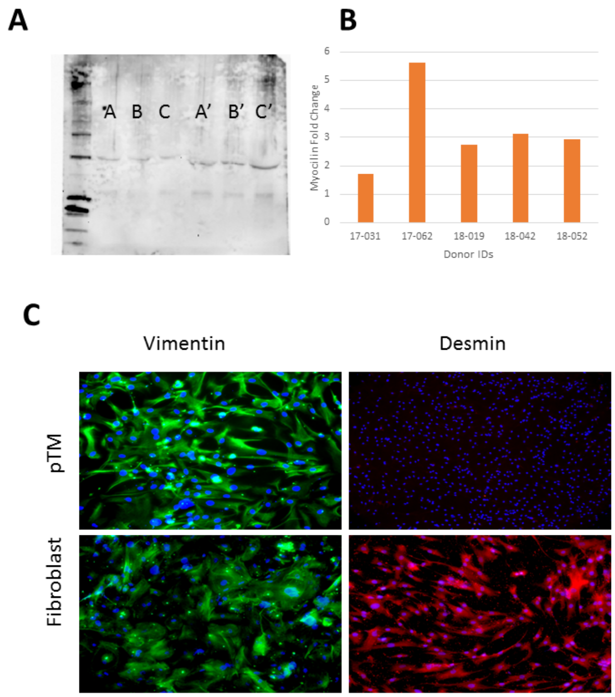

Verification of human primary TM lines

A- Representative Western blot of myocilin accumulation in cell culture media prior to dex stimulation (A, B, and C) and thereafter (A', B', and C'). The image provided depicts the unaltered full size blot. The image was neither cropped from the same gel or from other gels, fields or exposures.

B- Fold change of myocilin expression due to dex stimulation in the primary TM lines used in this study

C- Additionally all cell lines were screened for Vimentin expression (positive marker) and the absence of Desmin expression. Fibroblasts are shown as a control.
